# Effects of *Spartina alterniflora* invasion on the community structure and diversity of wetland soil bacteria in the Yellow River Delta

**DOI:** 10.1002/ece3.8905

**Published:** 2022-05-07

**Authors:** Shuai Shang, Shunxin Hu, Xiaoxue Liu, Yu Zang, Jun Chen, Ning Gao, Liangyu Li, Jun Wang, Longxiang Liu, Jikun Xu, Yumiao Zhang, Tao Wu, Xuexi Tang

**Affiliations:** ^1^ 117714 College of Biological and Environmental Engineering Binzhou University Binzhou Shandong China; ^2^ 12591 College of Marine Life Sciences Ocean University of China Qingdao Shandong China; ^3^ 605766 Shandong Provincial Key laboratory of Marine Ecological Restoration Shandong Marine Resource and Environment Research Institute Yantai China; ^4^ National Marine Environment Monitoring Center Dalian China

**Keywords:** invasion, soil microorganism, *Spartina alterniflora*, *Suaeda heteroptera*, wetland

## Abstract

The exotic plant *Spartina alterniflora* is expanding rapidly along China's coast regions, seriously threatening native ecosystems. Soil bacteria are important for biogeochemical cycles, including those of carbon, nitrogen, and sulfur, in wetland ecosystems. There is growing evidence that microorganisms are important in case of plant invasion. In the present study, we studied the interlacing area of *S*. *alterniflora* and *Suaeda heteroptera*, selected soil of invaded and non‐invaded regions and explored the effect of the composition and diversity of bacterial communities in coastal wetlands. The bacterial community composition of invasive and noninvasive areas was subjected to high‐throughput sequencing. In the five areas tested, the main bacterial phyla were Proteobacteria, Bacteroides, and Acidobacteria; the richness of the bacterial community in the soil increased after *S*. *alterniflora* invasion, most changes occurred at the genus level. The relative abundances of *Desulfobulbus* and *Sulfurovum* were higher in invasive areas than in noninvaded areas. PCA, RDA, and LEfSe analyses found that the *S*. *alterniflora* invasion significantly influenced the bacterial community and physicochemical properties of wetland soil. In conclusion, soil microbial community composition was tightly associated with *S*. *alterniflora* invasion. This study provide an important scientific basis for further research on the invasion mechanism of *S*. *alterniflora*.

## Graphical abstract

Soil microbes are important for biogeochemical cycles of carbon, nitrogen, and sulfur in wetland ecosystems. There is growing evidence that microorganisms are important for plant invasion. We explored the functional role of microorganisms during the invasion of *Spartina alterniflora* in coastal areas of China.

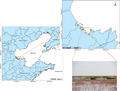

## 1 INTRODUCTION

Soil microbes play an important role in the formation, evolution, stability, and ecological function of coastal ecosystems and are closely related to the estuarine wetland environment and the elemental biogeochemical cycle (Boyle et al., 2008; Zhang, Hu, et al., 2017; Zhang, Nie, et al., 2017). All plants’ exudates affect microbes, but the exudates and abscisins released from invading plants can cause changes in the soil's physicochemical properties and shape the structure of the soil microbial community (Zhang, Bai, et al., 2020; Zhang, Liu, et al., 2020). For example, to adapt to the new environment, invasive species need to improve their adaptability and reproductive ability. Some invasive plants may accumulate pathogens more harmful to competitors to complete their invasion (Duchesneau et al., 2021). If invasive plants associate with fewer pathogens than native plants, they will have an advantage (Bickford et al., 2020). Thus, it is important to explore the role of the bacterial community in the invasion process.

As a perennial halophyte, *S*. *alterniflora* has a strong ecological adaptability and breeding ability (Zhang, Hu, et al., 2017; Zhang, Nie, et al., 2017). *S*. *alterniflora* spreads and grows in a large area of the coastal wetlands of China, on the niche of local species affecting the environment of the invaded land and forming a single dominant community (Nie et al., 2009; Subudhi & Baisakh, 2011). This way, it reduces local biodiversity destroying the structure and function of the original ecosystem. For example, a recent meta‐analysis found that plant invasion could alter rhizosphere microbial communities, particularly by increasing nitrogen mineralization, extracellular enzyme activity, and the abundance of arbuscular mycorrhizal fungi as well as reducing the abundance of soil pathogens and herbivores (Zhang et al., 2019), and another study found that plant invasion strongly changed the soil microbial community structure and composition of mangrove wetlands (Min et al., 2017). Particularly, Zheng explored the effect of *S*. *alterniflora* invasion on the rhizospheric bacterial community of mangrove wetlands and the control factors of soil function (Zheng et al., 2019). The soil bacterial and play an important role in invasion of *S*. *alterniflora* (Callaway et al., 2004; Gao et al., 2019; Zhang, Bai, et al., 2019). Therefore, it is necessary to assess the influence of invasive plants on soil microbes.

Coastal wetlands play a vital role in maintaining biodiversity, conserving water, and improving animal and plant resources (Bianchi et al., 2013). Meanwhile, coastal wetlands are the most sensitive to global change. The ecological invasion of the Yellow River Delta coastal wetland by *S*. *alternaltern* was bidirectional to the sea and back to land. *S*. *alternaltern* expanded its territory by sexual reproduction consolidating its position by asexual reproduction, continuously limiting the distribution area of *S*. *heteroptera*. Previous studies focused on the mechanism of *S*. *alterniflora* invasion and its impact on biomass, elemental cycling, and governance measures in indigenous ecosystems (Wang et al., 2014), and on its influence on the soil microbial characteristics of different indigenous plant communities at a regional scale (Zhang, Bai, et al., 2019; Zheng, Li et al., 2019). New technological developments, especially the emergence of molecular biological techniques, such as high‐throughput sequencing, provides favorable conditions for studying soil microbial community structure and diversity. Soil microbes play an important role in alien plant invasion. Invasive plants can modify native soil microbial community; in turn, changes in soil microbes can result in a positive or negative effect on the competition between native and invasive plants (Ravichandran & Thangavelu, 2017). Therefore, considering these effects is beneficial to predict the invasion mechanism. In the present study, we studied the interlacing area of *S*. *alterniflora* and *S*. *heteroptera*, selected soil of invaded and noninvaded regions and investigated its effect on the composition and diversity of bacterial communities in coastal wetlands.

## 2 MATERIAL AND METHODS

### 2.1 Study areas and sampling points

**FIGURE 1:**
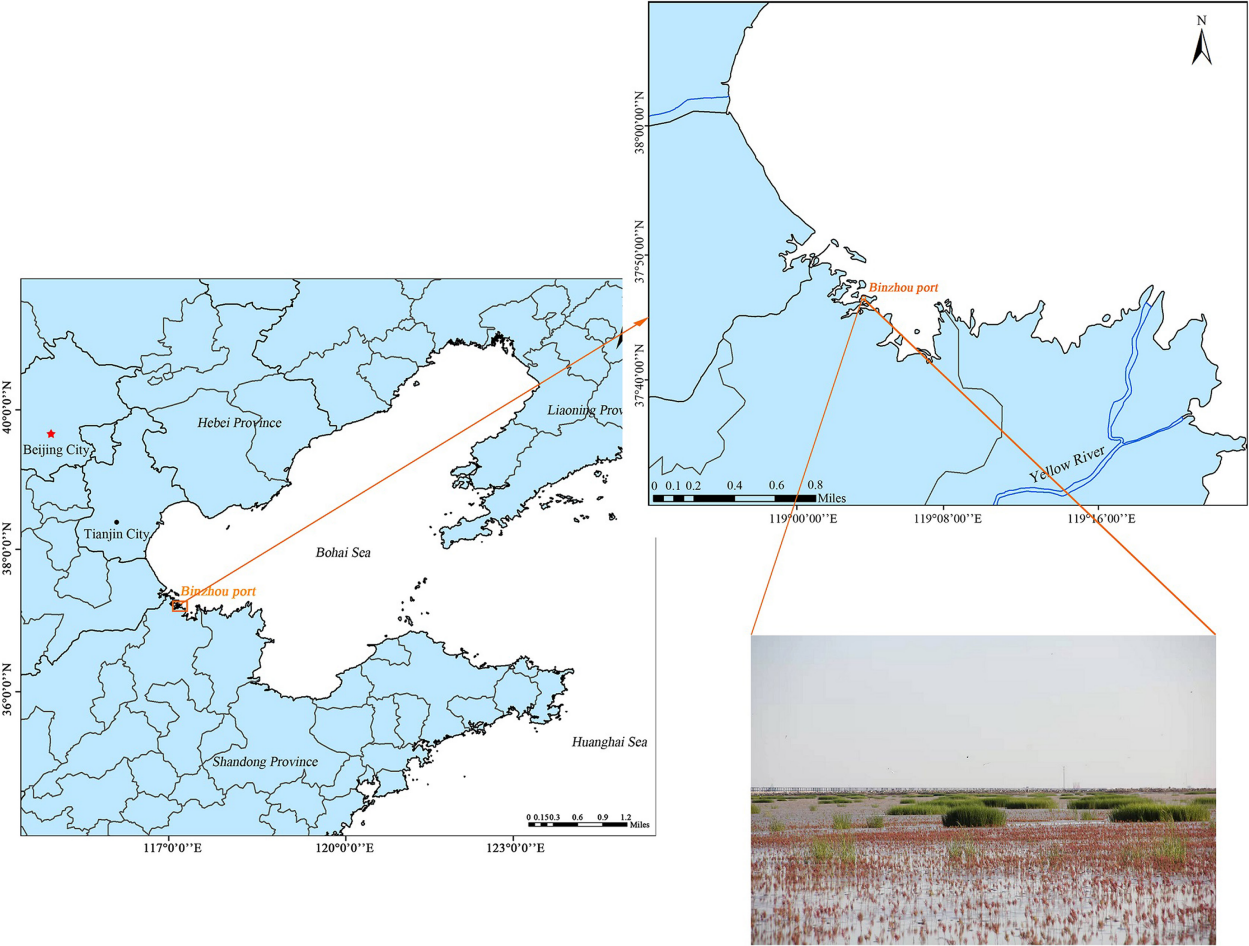
Sampling sites

The study area is located in the coastal wetland area of the Yellow River Delta (118.07°E, 38.18°N) which is shown in Figure 1. This area is characterized by a warm‐temperate continental monsoon climate. Its annual mean temperature is 11.5℃–12.4℃ and the annual mean rainfall is 530–630 mm^3^ (Zhang et al., 2021). In recent years, *S*. *alterniflora* expanded rapidly in the tidal flats of the Yellow River Delta. It occupied the habitat of native species becoming one of the dominant species (Ren et al., 2019). With the continuous invasion of *S*. *alterniflora*, the native species at the estuary's entrance have degraded to varying degrees. The vegetation community in the intertidal zone corresponds mainly to *S*. *heteroptera* (80–110 per square meter). Therefore, five sampling sites were set up in each experimental area to invade the intertidal pterine canopy wetland at the Yellow River Delta, we used the following samples (Table 1), including the *S*. *alterniflora* distribution region (Sa, MSoil, and Ssh groups), and the *S*. *heteroptera* distribution region (Ssw and WSoil groups).

**TABLE 1:** Information of different sample groups

Group	Samples name	Information
MSoil	MSoil_1	Bulk soil with invasion
	MSoil_2	Bulk soil with invasion
	MSoil_3	Bulk soil with invasion
	MSoil_4	Bulk soil with invasion
Sa	Sa_1	Rhizosphere soil of the *Spartina alterniflora*
	Sa_2	Rhizosphere soil of the *Spartina alterniflora*
	Sa_3	Rhizosphere soil of the *Spartina alterniflora*
	Sa_4	Rhizosphere soil of the *Spartina alterniflora*
Ssh	Ssh_1	Rhizosphere soil of the *Suaeda heteroptera* with invasion
	Ssh_2	Rhizosphere soil of the *Suaeda heteroptera* with invasion
	Ssh_3	Rhizosphere soil of the *Suaeda heteroptera* with invasion
	Ssh_4	Rhizosphere soil of the *Suaeda heteroptera* with invasion
Ssw	Ssw_1	Rhizosphere soil of the *Suaeda heteroptera* without invasion
	Ssw_2	Rhizosphere soil of the *Suaeda heteroptera* without invasion
	Ssw_3	Rhizosphere soil of the *Suaeda heteroptera* without invasion
	Ssw_4	Rhizosphere soil of the *Suaeda heteroptera* without invasion
WSoil	WSoil_1	Bulk soil without invasion
	WSoil_2	Bulk soil without invasion
	WSoil_3	Bulk soil without invasion
	WSoil_4	Bulk soil without invasion

### 2.2 Sample collection and processing

In November 2020, following the typicality and representativeness principle of sample layout and collection, a large sample area was determined in the mixed area of *S*. *heteroptera* and *S*. *alterniflora* growth before high tide. Five sampling sites were randomly set up in each sample area (four samples per plot). Before soil sampling, we removed visible plant residues from the soil surface. The five‐point sampling was used to collect each soil sample. A stainless steel soil collar was used to collect the soil samples. Bulk soils were taken between plant clusters to avoid the unreasonable impact of plant roots in each sampling site. The soil of a 0–10 cm soil layer was placed into sterilized self‐sealing bags, sealed with a portable incubator. At least 10 g root samples were collected from each *S*. *heteroptera* and *S*. *alterniflora* sample. The collected root samples were sealed and stored immediately in biological sample boxes for low‐temperature storage. The rhizosphere soil was collected in the laboratory. First, the bulk soils were plants were shaken off in the ultra‐clean workbench. Next, the soil within 1–2 mm of the root was collected with the brush and combined to form one composite soil sample per plot. Soil samples were placed in sterile plastic bags, and then divided into two subsamples and stored in a freezer at −80°C for subsequent DNA extraction and bacterial determination.

### 2.3 Environmental parameters

Soil samples from which visible plant litter and stones were removed, were collected using a stainless hand shovel. The soil samples were placed in polyvinylchloride bags. Each soil sample consisted of three replicates and was placed in a dry refrigerator and sent to the laboratory as quickly as possible. The pH of each soil sample was measured with a pH meter on the supernatant of a 1:5 soil‐water mixture (Sartorius PB‐10, Germany). The total organic carbon (TOC) of each soil sample was measured on a TOC analyzer (TOC‐L CPN; Shimadzu, Kyoto, Japan), the total nitrogen (TN) was determined on an Elemental Analyzer (CHOS, Elemental Analyzer, Vario EL, Germany), and the total sulfur (TS) was determined by inductively coupled plasma atomic emission spectrometry (ICP/AES).

### 2.4 Soil bacterial community structure analysis and high‐throughput sequencing technology

Genomic DNA was extracted from soil samples using the E.Z.N A. Soil DNA Kit and DNA purity and concentration were detected by agarose gel electrophoresis. The 16S V3–V4 region was amplified using 341F (5'‐CCTAYGGGRBGCASCAG‐3') and 805R (5'‐GACTACNNGGGTATAAT‐3') (Muhling et al., 2008). PCR reactions were performed in triplicate in 50‐μl mixtures containing 5 μl of 10 × KOD Buffer, 5 μl of 2.5 mM dNTPs, 1.5 μl of each primer (5 μM), 1 μl of KOD polymerase, and 100 ng of template DNA. PCR amplification conditions were 95°C for 2 min, followed by 27 cycles at 98°C for 10 s, 62°C for 30 s, and 68°C for 30 s and a final extension at 68°C for 10 min. To exclude false‐positive PCR results, negative control PCR products were purified with AMPure XT beads (Beckman Coulter Genomics, Danvers, MA, USA) and quantified with Qubit (Invitrogen, USA). Amplicons were pooled in equal amounts and were paired‐end sequenced on the NovaSeq PE250 platform, following standard protocols. The sequencing was conducted at Lc‐Bio Technologies Co., Ltd (Hangzhou, Zhejiang Province, China).

### 2.5 Data processing and analyses

Samples were sequenced on the Illumina NovaSeq platform as recommended by the manufacturer. We assigned the paired‐end sequence to the sample according to its unique bar code. Then, the bar code and primer sequences were removed. According to fqtrim (v.0.94), the quality filtering of raw read data was set to allow for high‐quality clean labels. The Vsearch software was used to filter chimeric sequences (v.2.3.4) (Rognes et al., 2016). Demodulation was performed using DADA2 (Benjamin et al., 2016), to obtain the feature table and sequence. The diversity was calculated by normalizing to the same random sequence. According to the SILVA (release 132) classifier, the characteristic abundance was normalized using the relative abundance of each sample. Then, the alpha diversity was used to analyze the complexity of sample species diversity with five indicators (Chao1, observed species, good coverage, Shannon, and Simpson). These indicators were calculated using QIIME2 (Beiko et al., 2018). Beta diversity was calculated using QIIME2. The Linear discriminant analysis effect size (LEfSe) was used to identify indicator bacterial groups in different sampling sites (Segata et al., 2011).

### 2.6 Statistical analysis

The Kruskal–Wallis test was used to determine significant differences in environmental factors among sampling sites. One‐way analysis of variance was used to analyze the abundance of microbial communities and environmental parameters. The Spearman's correlation coefficient was used to explore the relationships between the bacterial communities and environmental factors. IBM SPSS Statistics 19.0 for Windows was used to perform the ANOVA and correlation analysis. All data on soil physicochemical properties were standardized before PCA and RDA analysis. The RDA and PCA were conducted using the package in R v3.4.1.

## 3 RESULTS

### 3.1 Soil physicochemical properties

**FIGURE 2:**
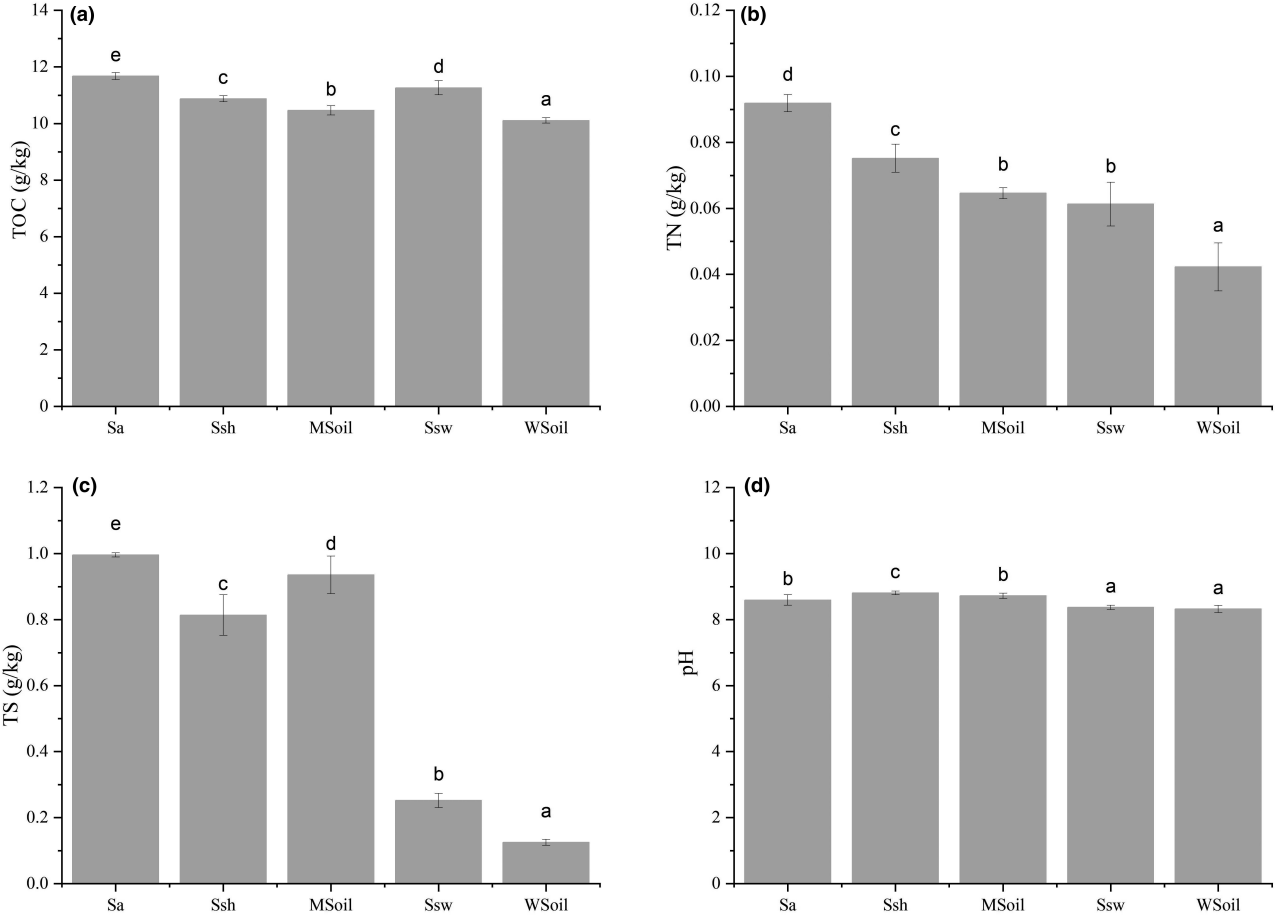
Soil physicochemical properties

Four soil physiochemical properties (TOC, TN, pH, and TS) of the sampling sites are shown in Figure 2, as are the significance indexes for comparison between groups. The results show that the TS and TOC of the sampling sites significantly differed among groups (*p* < .05). The TOC was significantly higher in the Sa group than in all other groups (*p* < .05). The TS was significantly higher (*p* < .05) in the invaded area (Sa, Ssh, and MSoil groups) than that of the noninvaded area (Ssw and WSoil groups). The pH was significantly higher in the Ssh and MSoil groups than in the Sa group (*p* < .05). In addition, the TN was significantly higher in the invaded area (Sa, Ssh, and MSoil groups) than in the WSoil (*p* < .05).

**FIGURE 3:**
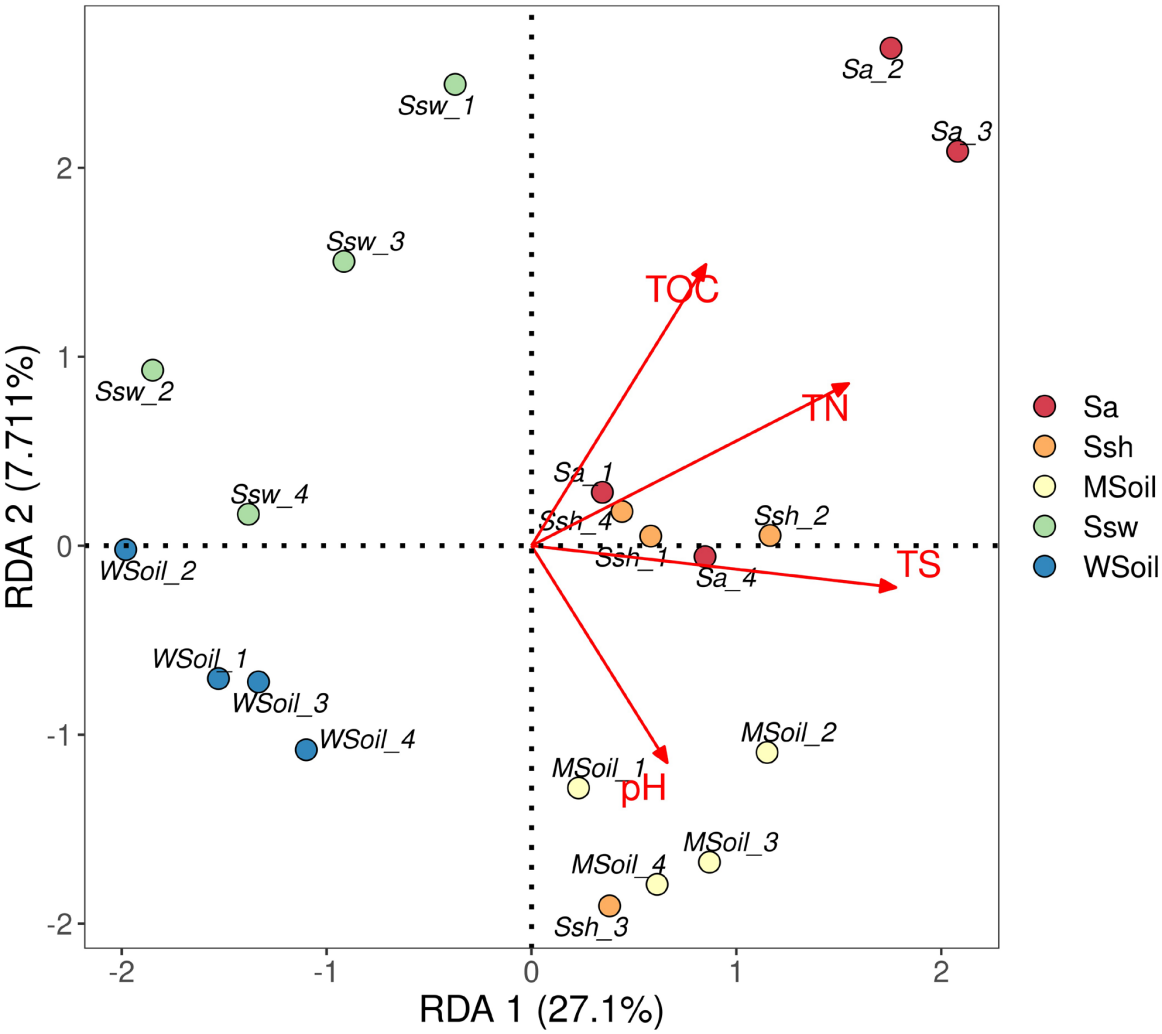
RDA between the bacterial community composition and the soil's physicochemical properties in wetland soil

The RDA results showed that the relationship between the composition of the bacterial community and soil physicochemical properties in wetland soil changed after *S*. *alterniflora* invasion. And the first two RDA axes are 0.0711 and 0.271, respectively. The degree of variation in bacterial community composition was 34.8% (Figure 3).

### 3.2 Diversity analysis of soil bacteria

**FIGURE 4:**
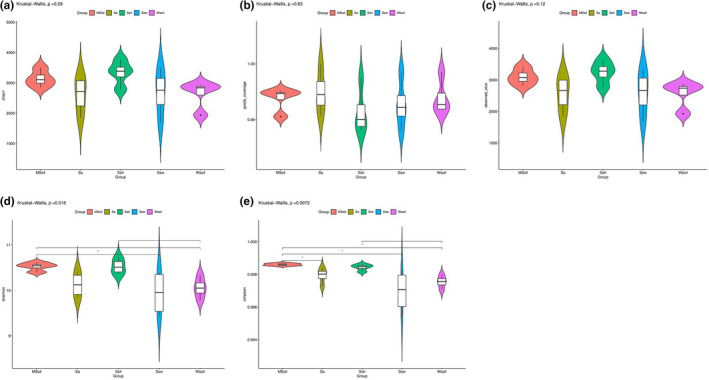
Soil bacterial diversity index for each sample

The Shannon index (*p* < .05) exhibited significant differences among sampling sites (Figure 4), being significantly lower in the Sa and Ssw groups. The coverage of all samples was >0.99, indicating that the sequencing depth was sufficient to cover most microorganisms (Figure 4).

**FIGURE 5:**
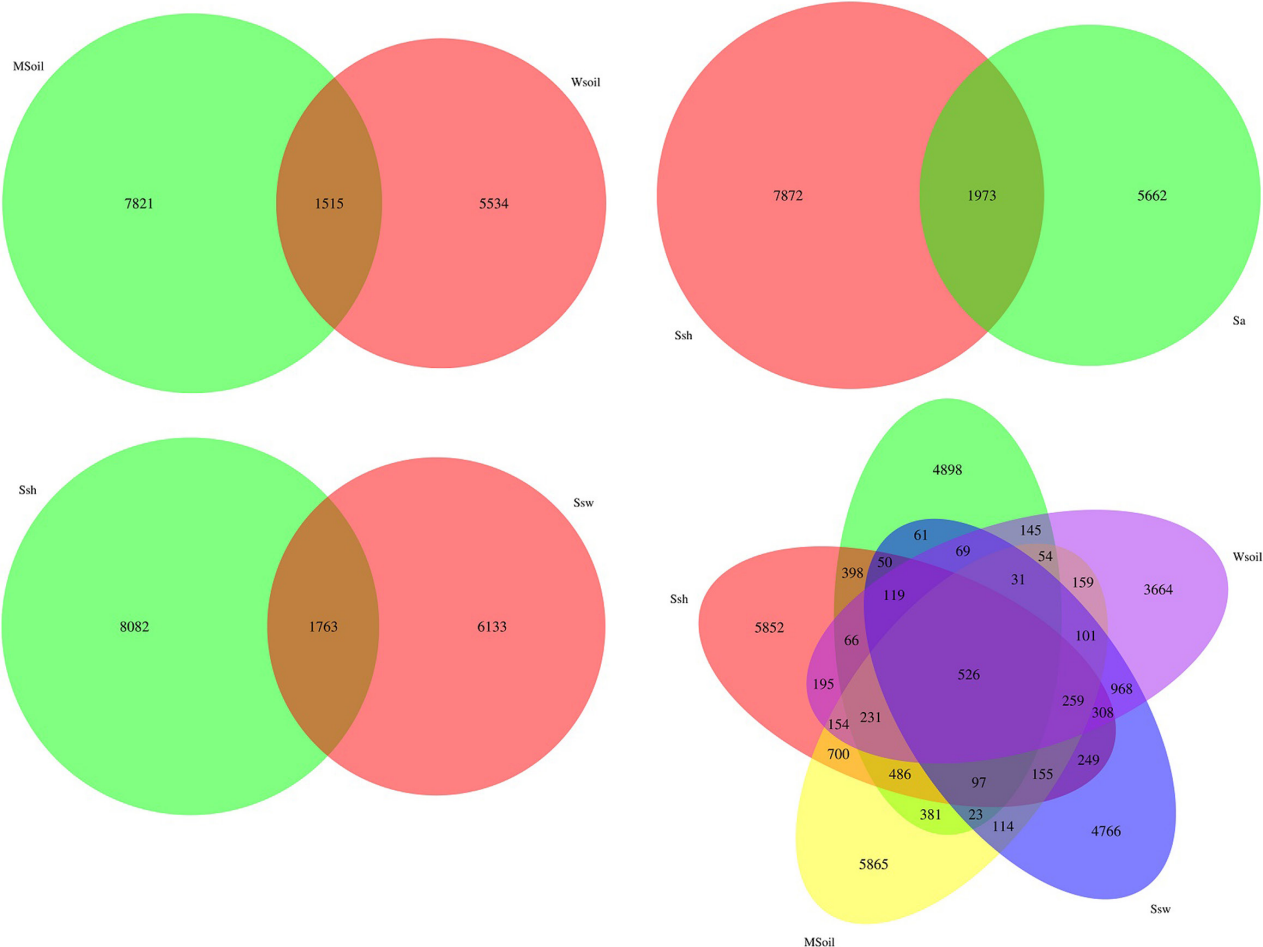
Venn diagram showing the number of shared and unique bacterial operational taxonomic units (OTUs) among all samples. Values within intersections represent the number of shared OTUs and values outside represent the number of unique OTUs. MSoil, bulk soil from the invaded site; Sa, *S*. *alterniflora* rhizosphere soil; Ssh, *S*. *heteroptera* rhizosphere soil from the invaded site; Ssw, *S*. *heteroptera* rhizosphere soil from the noninvaded site; WSoil, bulk soil from the noninvaded site

Over 9845 OTUs were identified in the *S*. *heteroptera* rhizosphere soil in the invaded area, and 7896 OTUs were identified in the S. *heteroptera* rhizosphere soil in the noninvaded area (Figure 5). There were 9336 OTUs identified in the invaded bulk soil and 8049 OTUs identified in the noninvaded bulk soil. A total of 7872 and 5662 OTUs were identified in the *S*. *heteroptera* and *S*. *alterniflora*, respectively.

### 3.3 Composition of the bacterial community

**FIGURE 6:**
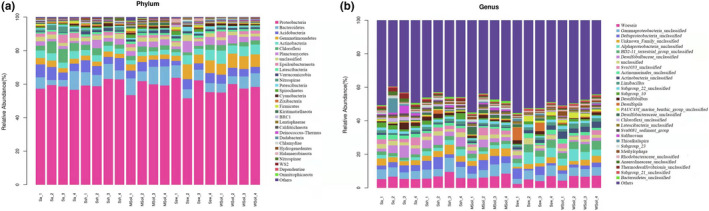
Relative abundance of bacterial (a) phylum and (b) genus of different samples. MSoil, bulk soil from the invaded site; Sa, *S*. *alterniflora* rhizosphere soil; Ssh, *S*. *heteroptera* rhizosphere soil from the invaded site; Ssw, *S*. *heteroptera* rhizosphere soil from the noninvaded site; WSoil, bulk soil from the noninvaded site

Based on species annotation, we selected the top 10 species with maximum abundance at the phylum level for each sample. The phylum‐level composition is shown in Figure 6. The predominant phyla in the five groups were Proteobacteria, Bacteroidetes, and Acidobacteria, accounting for >70% of all identified bacteria. Our results showed that the invasion did not significantly change the composition of the dominant bacterial phyla. However, it did change the relative abundance of Gemmatimonadetes.

**FIGURE 7:**
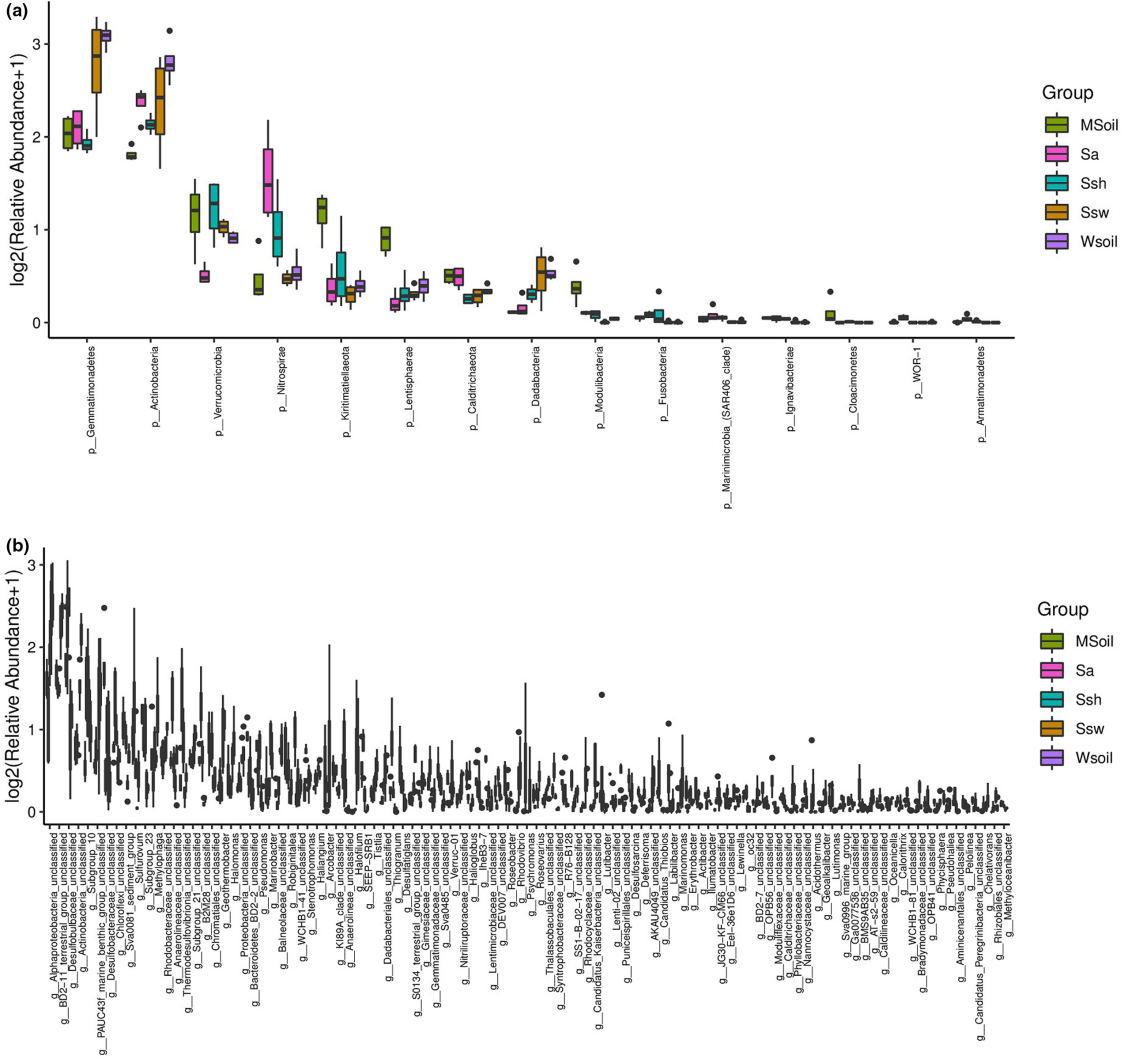
Boxplot of bacterial community composition at phylum (a) and genus (b) level

In this study, abundant bacteria were detected at the genus level (Figure 6). *Woeseia* was dominant in all groups, whereas the community composition of *Desulfobulbus* was different. The community composition of *Desulfobulbus* and *Sulfurovum* was higher in the invaded area than in the separated area (Figure 7).

### 3.4 Principal component analysis of bacterial community composition

**FIGURE 8:**
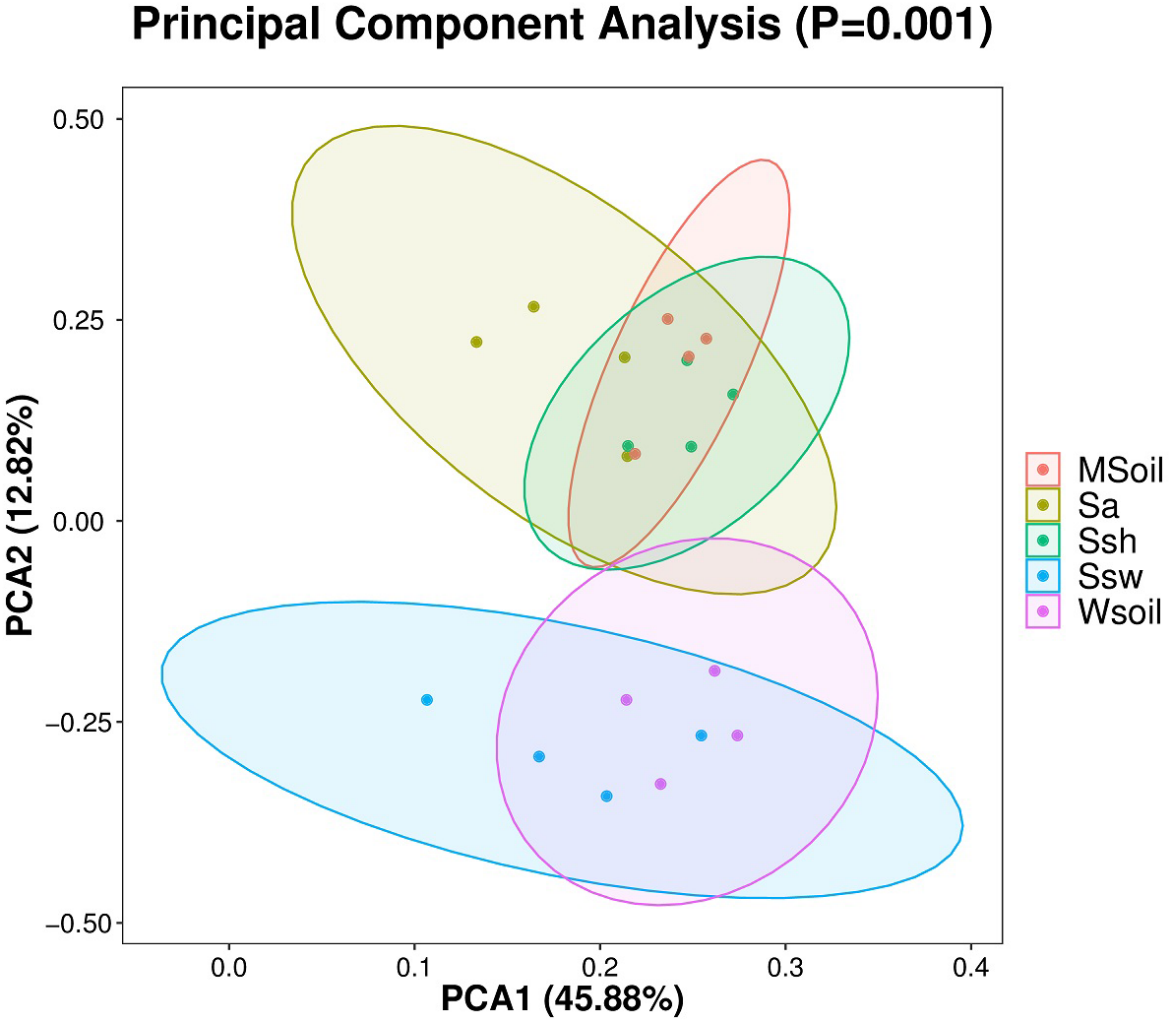
Principal component analyses of the bacterial community composition in different samples. Ellipses represent the sampled compartments. MSoil, bulk soil from the invaded site; Sa, *S*. *alterniflora* rhizosphere soil; Ssh, *S*. *heteroptera* rhizosphere soil from the invaded site; Ssw, *S*. *heteroptera* rhizosphere soil from the noninvaded site; WSoil, bulk soil from the noninvaded site

PCA was performed to reveal the influence of invasion on the soil bacterial community. As shown in Figure 8, the cumulative contribution rate of variance of the first two principal components extracted was 58.70%, which indicated that they were the main contributors to differences in the bacterial community before and after *S*. *alterniflora* invasion. PCA analysis showed that the changes in the bacterial community of the invaded and noninvaded areas of *S*. *alterniflora* were greatly affected by these two principal components.

### 3.5 LEfSe analysis

**FIGURE 9:**
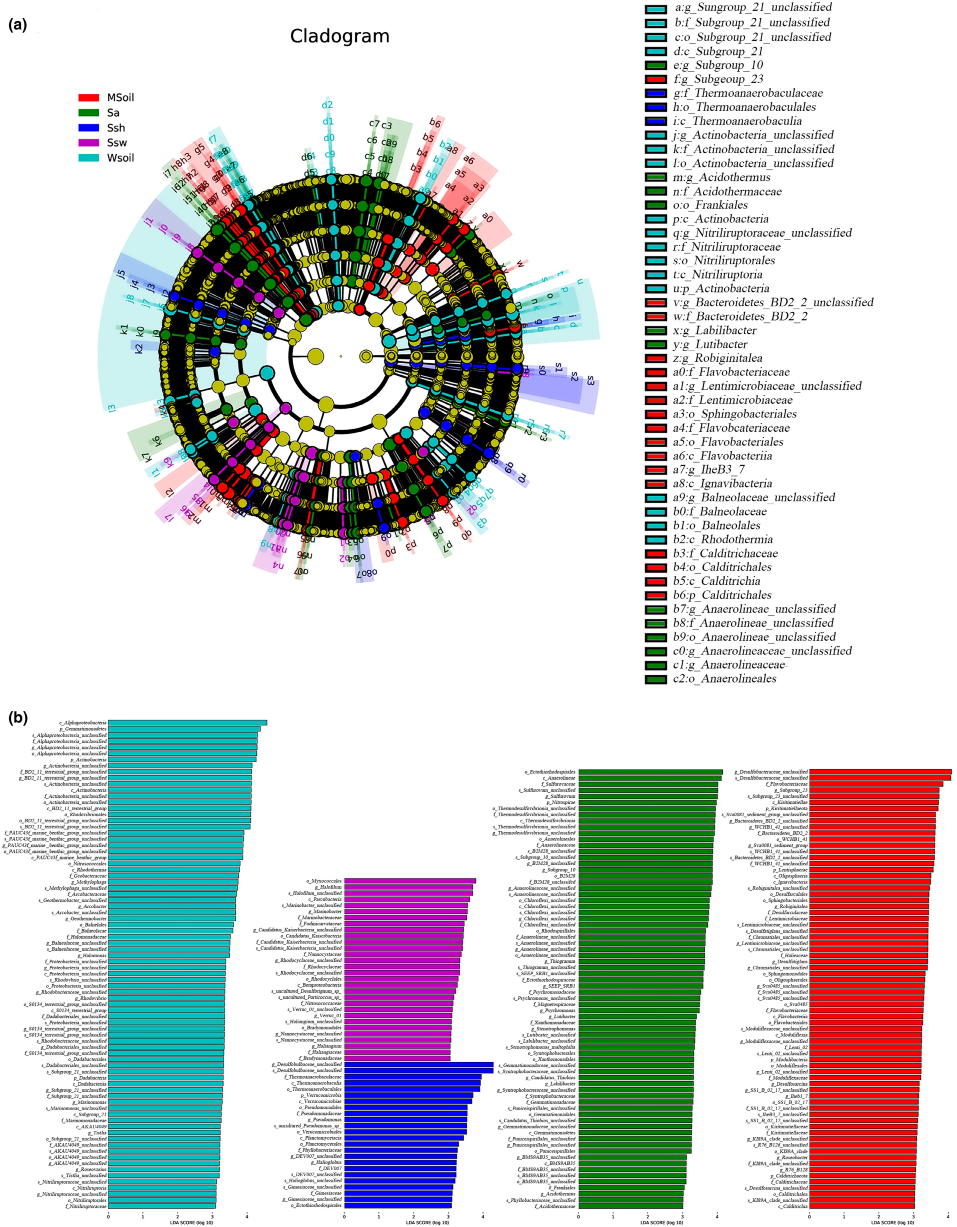
Indicator bacterial groups associated with the five groups. LEfSe (LDA effect size) was used to compare ≥2 groups to determine the species showing significant differences in abundance between groups. MSoil, bulk soil from the invaded site; Sa, *S*. *alterniflora* rhizosphere soil; Ssh, *S*. *heteroptera* rhizosphere soil from the invaded site; Ssw, *S*. *heteroptera* rhizosphere soil from the noninvaded site; WSoil, bulk soil from the noninvaded site

In this study, we first compared species composition between groups (Figure 9). Our results showed that Gemmatimonadetes and Actinobacteria (at the phylum level), and *Methylophaga*, *Arcobacter*, *Geothermobacter*, *Halomonas*, *Rhodovibrio*, *Marinomonas*, *Tistlia*, and *Roseovarius* (at the genus level) were dominant in soil without invasion, as were *Halofilun*, *Marinobacter*, *Verruc*_1, *Haliangium* (at the genus level) in the *S*. *heteroptera* soil without invasion. *Verrucomicrobia* (at the phylum level), *Pseudomonas*, and *Halioglobus* were dominant in the *S*. *heteroptera* with invasion, and Nitrospirae (at the phylum level), *Sulfurovum*, *Thiogranum*, *Psychromonas*, *Lutibacter*, *Stenotrophomonas*, *Candidatus_Thiobios*, and *Labilibacter* were dominant in the *S*. *alterniflora* rhizosphere. Kiritimatiellaeota, Lentisphaeria, Modulibacteria, and Calditrichaeota (at the phylum level), as well as *Robiginitalea*, *Desulfatiglans*, *Desulfosarcina*, and *Roseobacter* (at the genus level) were dominant in the soil with invasion (Figure 9).

**FIGURE 10:**
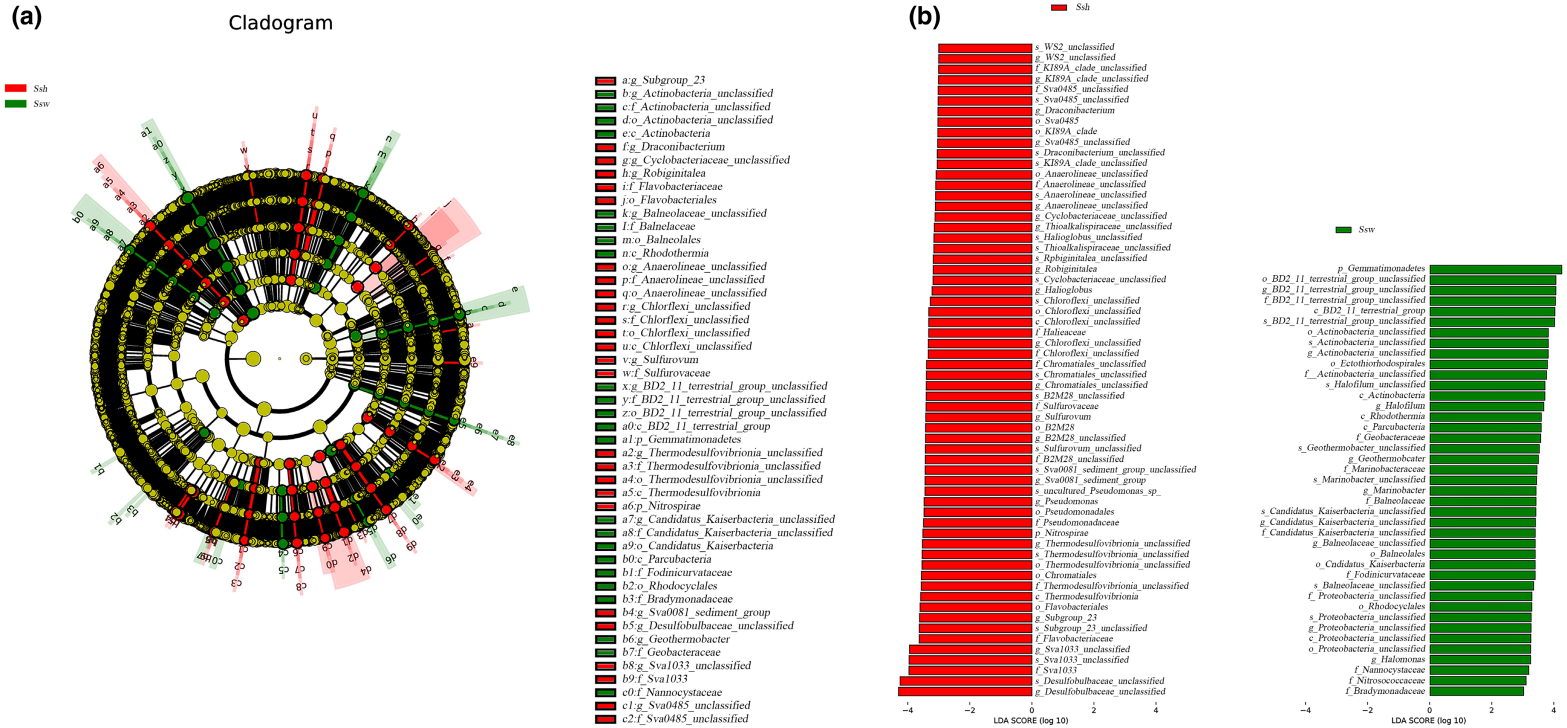
Indicator bacterial groups associated with two groups. LEfSe (LDA effect size) was used to compare ≥2 groups to determine the species showing significant differences in abundance between groups. MSoil, bulk soil from the invaded site; Sa, *S*. *alterniflora* rhizosphere soil; Ssh, *S*. *heteroptera* rhizosphere soil from the invaded site; Ssw, *S*. *heteroptera* rhizosphere soil from the noninvaded site; WSoil, bulk soil from the noninvaded site

LEfSe analysis was used to identify the different species between the invaded and noninvaded areas of *S*. *heteroptera* (Figure 10). Our results showed that levels of Gemmatimonadetes (at the phylum level), *Halofilun*, *Marinobacter*, and *Halomonas* (at the genus level) were significantly different in the noninvaded area of *S*. *heteropteran*, and Nitrospirae (at the phylum level), *Draconibacterium*, *Robiginitalea*, *Sulfurovum*, and *Pseudomonas* were identified as significantly different present species in the invaded area of *S*. *heteroptera* (Figure 10).

## 4 DISCUSSION

We investigated the soil bacterial communities in the *S*. *alterniflora* community, *S*. *heteroptera* and *S*. *alterniflora* mixed community, and *S*. *heteroptera* community in the Yellow River Delta. Soil microbial communities are influenced by a variety of biotic and abiotic factors (Gao et al., 2019; Li et al., 2021). In a stable ecosystem, plants and soil microorganisms form a balanced state of symbiosis in long‐term development. Plant species can release certain degradation products and exudates to the soil, which provide nutrients for soil microorganisms further affecting the diversity and composition of soil microbial communities (Wang, Fang, et al., 2021). Furthermore, soil microorganisms are sensitive to the soil environment, such that community structure and diversity are influenced by soil environmental factors (Delgado‐Baquerizo et al., 2016). As a result, when *S*. *alterniflora* replaced the vegetation, it modified the quality and quantity of products entering the soil, changing its physicochemical properties and affecting soil microbial communities.

Our results showed that soil bacterial diversity indices (Shannon and Simpson indices) differed significantly between invaded and noninvaded areas. The number of unique and shared OTUs in the invaded area was significantly higher than in the noninvaded area. Previous studies reported that compared with native species, *S*. *alterniflora* has a more developed root system and higher net primary productivity, which would provide more available substrates for the growth of soil microorganisms (Wang, Yuan, et al., 2021; Yuan et al., 2015), however, a previous study showed a decline in bacterial diversity over time of invasion (Zhang, Bai, et al., 2020; Zhang, Liu, et al., 2020). In addition, the strong root system of *S*. *alterniflora* root system can loosen the soil and improve the soil's gas supply and pH value, thus creating a rich and diverse microenvironment in the soil. This increased soil pH can help release the dissolved organic matter and improve bacterial diversity (Qu et al., 2020). Therefore, the increased diversity and richness of soil bacterial communities can be explained by increasing available nutrients and the improved soil environment resulting from *S*. *alterniflora* invasion.

Similar to previous studies, our results showed that the main bacterial phyla in the five areas were Proteobacteria, Bacteroides, and Acidobacteria (Qu et al., 2020; Yu et al., 2012). Proteobacteria was the most predominant group; the most ubiquitous and common group in the soil due to its rapid growth and adaptation (Islam et al., 2020). Thus, the invasion did not significantly change the community composition of the dominant bacterial phyla due to the similar vegetation growth before and after, however, the invasion influenced the relative abundance of Gemmatimonadetes. Gemmatimonadetes is the least known among the seven known phototrophic phyla, and its function remains unknown (Zeng et al., 2016). Therefore, this change in Gemmatimonadetes before and after invasion requires further research.

Because of the invasion, the bacterial communities differed at the genus level. Compared with the invaded area, the *Limibacillus* and *Methylophaga* were more abundant in the noninvaded area. However, the relative abundance of *Desulfobulbus* and *Sulfurovum* was higher in the invaded than noninvaded area. *Desulfobulbus* is known as sulfate‐reducing bacteria (SRB). A study demonstrated that its abundance increased following *S*. *alterniflora* invasion because of substrate stimulation of sulfate reducers from root exudates (Zeleke et al., 2013). SRB can utilize organic matter as an electron donor and sulfate as an electron acceptor to produce hydrogen sulfide (H_2_S). H_2_S is released into the surrounding area, directly or indirectly harming other vegetation in the invaded area. Additionally, SRB reduce sulfate to sulfides that coexist with iron, thereby reducing iron availability for the vegetation. The invasion of *S*. *alterniflora* has been shown to affect wetland ecosystems by changing the functional microbial community and affecting carbon, nitrogen, and sulfur cycles (Liao et al., 2007). The reducing bacteria in the rhizospheric soil of *S*. *heteroptera* provide the appropriate conditions for the invasion of *S*. *alterniflora*. This is also supported by the fact that *S*. *alterniflora* could change the abundance of the microbial taxa associated with the soil environment, thus promoting its rapid expansion and invasion; consistent with studies on *Mikania micrantha* (Li et al., 2006) and *Ageratina adenophora* (Zou et al., 2006).

LEfSe was used to compare between ≥2 groups of the main target and find significant differences in abundance between groups. Nitrospirae was identified as divergent species in the *S*. *alterniflora* rhizosphere at the phylum level. Nitrospirae is a group of Gram‐negative bacteria which can oxidize nitrite into nitrate (Han et al., 2020). Furthermore, the rhizosphere microbes of *S*. *heteroptera* were affected by the invasion. *Marinobacter* and *Halomonas* were reduced by the invasion area of *S*. *alterniflora*. *Marinobacter* are one of the most ubiquitous bacteria in the world's deep oceans, coastal sediments, etc. Most species are involved in nitrate reduction, and there is also evidence for Fe (III) reduction and metal (loid) detoxification. A study also found that *Marinobacter* species might perform an important and underestimated role in the biogeochemical cycling of organics and metals in varied marine and coastal habitats (Handley & Lloyd, 2013). *Halomonas* has salt and alkali resistance characteristics which play an important role in plant stress resistance. We speculated that the decrease in the two bacteria genera caused by *S*. *alterniflora* invasion is not conducive to the stress resistance of *S*. *heteroptera*, but will provide favorable conditions for *S*. *alterniflora* invasion. We found that *Sulfurovum* was increased in the invaded area of *S*. *heteroptera* after *S*. *alterniflora* invasion. *Sulfurovum* probably plays an important role in the carbon, sulfur, and nitrogen cycles of coastal and marine environments (Sun et al., 2020). Compared with *S*. *heteroptera*, *Sulfurovum* was mainly enriched in *S*. *alterniflora* invaded area*s*, resulting in sulfate accumulation. We suggest that the increase in *Sulfurovum* is a response to invasion. Interestingly, Nitrospirae increased in the invaded area of *S*. *heteroptera* after *S*. *alterniflora* invasion. Compared with *S*. *heteroptera*, Nitrospirae was enriched in *S*. *alterniflora*, acting as a self‐preservative for *S*. *heteroptera* in the competition for carbon sources. This aspect of the research is an important scientific evaluation of the invasion of *S*. *heteroptera* on the soil function of coastal wetlands. Therefore, a better understanding of plant–microbial interactions and the differences between native and invasive plants will contribute to our overall understanding of plant invasion mechanisms.

## 5 CONCLUSION

The response of soil bacterial communities in the Yellow River Delta after the invasion of *S*. *alterniflora* was investigated in this study. We determined that soil microbial community composition was tightly associated with the invasion of *S*. *alterniflora*. This study could serve as a basis for further research on the invasive mechanisms of *S*. *alterniflora*.

## CONFLICT OF INTERESTS

The authors declare no competing interests.

## AUTHOR CONTRIBUTIONS

**Shuai Shang:** Funding acquisition (lead); Methodology (lead); Writing – original draft (lead). **Shunxin Hu:** Formal analysis (equal); Writing – original draft (equal). **Xiaoxue Liu:** Writing – review & editing (equal). **Yu Zang:** Data curation (equal); Software (equal). **Jun Chen:** Methodology (equal); Validation (equal). **Ning Gao:** Investigation (equal); Software (equal). **Liangyu Li:** Data curation (equal); Writing – review & editing (equal). **Jun Wang:** Investigation (equal); Software (equal). **Longxiang Liu:** Methodology (equal); Writing – review & editing (supporting). **Jikun Xu:** Methodology (supporting); Software (supporting). **Yumiao Zhang:** Methodology (supporting); Writing – original draft (supporting). **Tao Wu:** Data curation (equal); Writing – review & editing (supporting). **Xuexi Tang:** Funding acquisition (equal); Writing – review & editing (equal).

## Data Availability

All sequences analyzed in the present study can be assessed in the SRA database under the accession number (SAMN17765397‐17765417) and the Zenodo (https://doi.org/10.5281/zenodo.6475327).
